# Corneal Extracellular Vesicles: Small Packages with a Big Impact

**DOI:** 10.3390/pharmaceutics18020186

**Published:** 2026-01-31

**Authors:** Brenna S. Hefley, Pawan Shrestha, Tina B. McKay, Peter Nsiah, Yasamin Moradi, Sarah E. Nicholas, Dimitrios Karamichos

**Affiliations:** 1 North Texas Eye Research Institute, University of North Texas Health, 3500 Camp Bowie Blvd, Fort Worth, TX 76107, USA; brenna.hefley@unthealth.edu (B.S.H.); pawanshrestha@my.unthsc.edu (P.S.); peternsiah@my.unthsc.edu (P.N.); yasaminmoradi@my.unthsc.edu (Y.M.); sarah.nicholas@unthealth.edu (S.E.N.); 2 College of Biomedical and Translational Sciences, University of North Texas Health, 3500 Camp Bowie Blvd, Fort Worth, TX 76107, USA; 3 Mass General Brigham Department of Anesthesiology, Massachusetts General Hospital, Boston, MA 02114, USA; tmckay@mgh.harvard.edu; 4 Department of Family Medicine, Texas College of Osteopathic Medicine, University of North Texas Health Science Center, 3500 Camp Bowie Blvd, Fort Worth, TX 76107, USA

**Keywords:** extracellular vesicles, exosome, therapeutics, characterization, biological fluid

## Abstract

Extracellular vesicles (EVs) are small membrane-bound particles that play a vital role in intercellular communication by facilitating the transfer of molecular cargo. In this review, we provide an overview of EV biology in corneal diseases, along with current approaches to therapeutic uses of EVs. Since EVs generally retain surface markers indicative of their cell of origin, they possess a degree of tissue specificity, which benefits drug delivery systems and highlights their potential as biomarkers to study disease processes. Further advances in technology and methodology will accelerate our understanding of EVs and help guide the field towards improved diagnostic techniques and therapeutic targets. We summarize EVs and their potential impact in medicine with a discussion of the limitations that remain in current approaches, as well as areas to focus on for future growth.

## 1. Introduction

When a child is born, they are introduced to a whole new world of sensations. There are now loud noises, cold environments, and having to breathe for the first time. The child opens their eyes, but the world is blurry due to their undeveloped retina. As the child grows, their eyesight improves day by day as the retina forms. Once the child can see clearly, visual perception becomes an essential part of their daily life. They experience the delight of seeing their parents’ faces and their favorite toy clearly, as well as dangers like stairs and obstacles. This person is also able to use the computer, drive, and experience a normal existence. But what if all of this were taken away due to corneal dystrophies?

**Figure 1:**
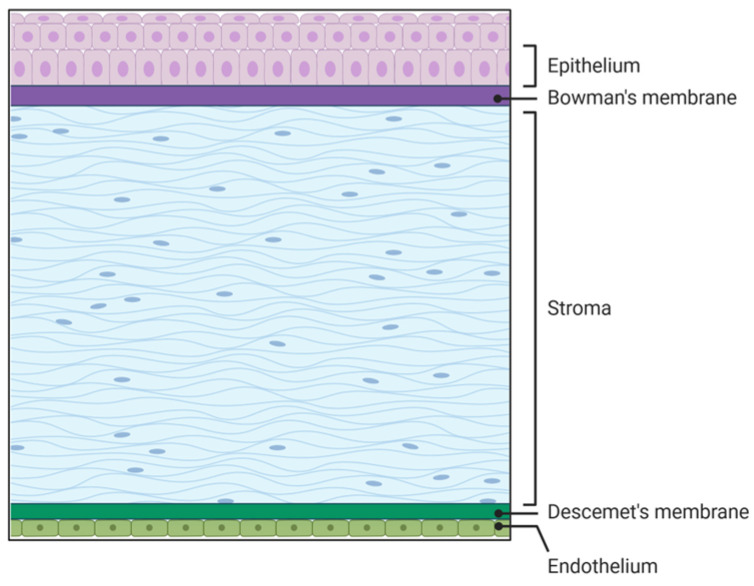
Five layers of the cornea. Source: Created in BioRender. Hefley, B. (2026) https://BioRender.com/r1otvqv.

The cornea is the transparent part of the eye located on the anterior segment of the eye. The human cornea plays a crucial role in vision by providing two-thirds of the refractive power of the eye [1,2]. It comprises three cellular and two acellular layers. The epithelial layer is the outermost corneal layer that can regenerate after being wounded in healthy individuals, but has delayed wound healing in diseased corneas such as those with diabetes [3,4,5]. The next layer is the acellular Bowman’s layer, which separates the epithelium from the stromal layer. The stromal layer is the thickest layer of the cornea and is made up of resident cells termed keratocytes as well as extracellular matrix (ECM) components like collagen, decorin, lumican, and keratocan, to name a few. The collagen fibrils run parallel to each other, which is essential for corneal transparency and enhances its refractive power [1]. The fourth layer is the Descemet’s membrane, which is the second acellular layer and acts as a barrier against infection. It divides the stromal layer from the endothelial layer, which is the final layer of the cornea that is in contact with the aqueous humor (Figure 1).

The cornea is a complex, highly innervated [6,7,8], and immune-privileged tissue [9,10,11] that requires cell–cell communication between the various layers. One structure that the cornea uses to achieve this communication is by using EVs.

EVs are membrane-bound particles that facilitate intercellular communication by passing cargo such as DNA, RNA, and proteins from one cell to another [12,13,14,15]. Here, we provide an overview of EV research in corneal dystrophies and discuss current approaches for potential therapeutic use. There is a growing interest in EVs as biomarkers of disease and drug carriers, which will also be discussed in this review.

## 2. Extracellular Vesicles (EVs)

**Figure 2:**
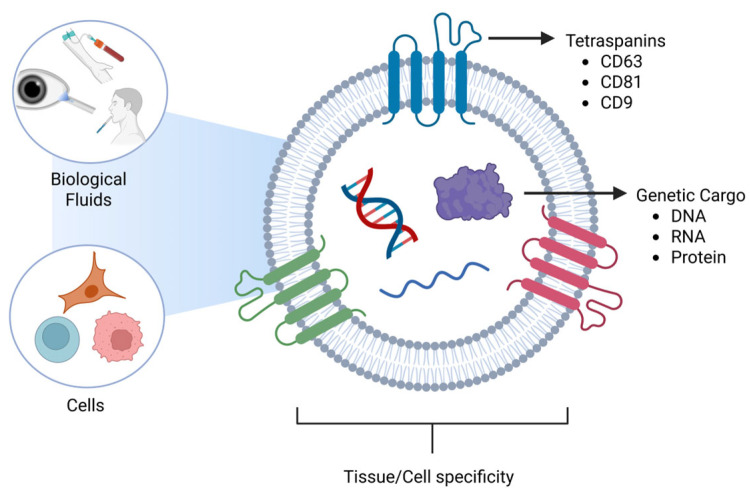
Schematic diagram of Extracellular vesicles with tetraspanins and genetic cargo in different biological fluids. Source: Created in BioRender. Hefley, B. (2026) https://BioRender.com/x18k556.

Early studies of EVs began in the 1960s [16], as EVs were initially thought to be vesicles for the release of unwanted cellular material. The history of their discovery and study has been well-reviewed in sentinel papers in the field [17,18,19]. There are three major categories of EVs: exosomes, microvesicles, and apoptotic bodies. Exosomes are the smallest EVs whose diameters range from 30–150 nm [20,21,22], and are produced by the endosomal membrane system [18,23,24]. Microvesicles are the intermediate vesicles, which range from 100 to 1000 nm [25,26,27] and are formed from the budding of the plasma membrane [24,28,29]. Apoptotic vesicles are the largest of the EVs, which are 1000–5000 nm [30,31,32] and also bud off the plasma membrane [31,32,33]. These size ranges do not definitively determine the subtype of EVs due to overlapping size ranges. There is a large debate in the field about the actual size range of exosomes, with some reports stating that they range between 30 nm and 150 nm [20,22,34], while other reports state a range of 50 nm–150 nm [35,36,37]. These differences in reporting the size can be due to several factors, such as isolation techniques or even the measuring equipment. While isolating the exosomes, different techniques, such as ultracentrifugation with its high speed, could cause the squishing of exosomes, making them smaller, while the gentler process, like size-exclusion chromatography (SEC), will provide integrity and preserve their size to 50–150 nm. Mitochondria-derived EVs are another subclass of EVs that originate from mitochondria and contain selective proteins, including TOM20 and OPA1, as well as mitochondrial DNA [38]. Intact extracellular mitochondria have also been detected in blood and in vitro [39,40]. Cancer research has expanded our knowledge of EVs by discovering that they not only travel short distances between cells, but they can also travel large distances in the body via the bloodstream [41,42,43]. EVs have been found in various biological fluids such as blood [44,45,46], tears [47,48,49], saliva [50,51,52], breast milk [53,54,55,56,57,58], and cerebrospinal fluid, to name a few (Figure 2).

EVs can be isolated by utilizing various methods such as ultracentrifugation, flow cytometry, size exclusion chromatography (SEC), exosome precipitation, and microfluidics-based isolation techniques [59,60]. Regular centrifugation is not sufficient in isolating EVs due to their small nature. This process requires the user to centrifuge their samples at various speeds to remove cells and cell debris, and then centrifuge the supernatant at an even higher speed to pellet the EVs [61]. Ultracentrifugation is the most widely used method for EV isolation [62], but prolonged use of the centrifuge can alter the shape of the EVs [27]. Ultrafiltration is one of the popular size-based exosome isolation methods along with SEC. It does not require special equipment, as membrane filters can be used with defined molecular weight or size exclusion limits, making it faster compared to ultracentrifugation [59].

Flow cytometry is another commonly used method to isolate EVs, which has the advantage of evaluating EVs at multiple time points and end points, as well as single EV analysis. This method has some limitations, in which the EV signal can be weak [63] compared to ultracentrifugation, and can cause the clogging of the filter pores in the flow cytometer [64]. Microfluidics-based isolation is considered to be the next generation of exosome research, as it uses precision engineering in manipulating tiny volumes of fluids using the physical and biochemical properties of exosomes [59]. Despite having their advantages, all these methods of isolation have their own limitations based on their unique characteristics and cause low to moderate purities in isolated exosomes.

Technological advances in recent years have markedly improved the methods available for EV phenotyping. Super-resolution microscopy [65] and fluorescent tags that are covalently bound to the EV membrane have enabled imaging and tracking of EVs at or near the single-particle level in vitro and in vivo [66,67]. Of these methods, fluorescent reporters (red tdTomato or GFP) mediated via palmitoylation permit dynamic monitoring of EVs that are derived from the host cell plasma membrane and secreted into the extracellular space [68]. Cell-type-specific markers have been used to enrich for neuronal-derived EVs found in blood based on neural cell adhesion molecules (NCAM or LCAM), allowing for the study of acute changes occurring in the central and peripheral nervous systems [69,70]. Nanoparticle tracking analysis (NTA) can also be used to measure the size and total EV concentration of the sample [71,72,73]. EV cargo can be analyzed by isolating the vesicle and applying molecular methods, such as polymerase chain reaction [74,75,76] (PCR) and western blotting [77,78,79] to analyze the genetic content.

EVs may be tissue-specific due to proteins located on their surface [80]. One group of proteins that is heavily studied in EV research is tetraspanins, which span the plasma membrane four times. The three tetraspanins commonly observed in EVs are CD63, CD81, and CD9. In the body, CD63 is located in the endosomal membrane system [81,82,83] and plays a role in the immune system, as well as binding and sorting cholesterol into endosomes for storage and distribution via EVs [84,85,86]. CD81 and CD9 play a role in inflammation [87,88], cell growth [89,90], cell proliferation [91,92], and are located on the plasma membrane [88,93].

### EV Heterogeneity

Although EVs can be produced from the same cell type, for example, fibroblasts, the EVs can experience heterogeneity, which can alter the surface protein expressions as well as the molecular cargo [94]. The changes in the molecular cargo can give insight into the pathological and physiological state of their cell of origin, which allows tracking of disease progression [94]. EVs have been shown to carry multiple kinds of DNA, which include double-stranded DNA, single-stranded DNA, mitochondrial DNA, and circular DNA. This cargo can then be transferred horizontally to other cells, which promotes genetic diversity but can also influence a healthy cell to develop a diseased phenotype.

EV heterogeneity is vastly understudied, and further studies are warranted in this field. Mastering this concept could lead to developing EVs for clinical trials as well as mass production of therapeutics.

## 3. EVs in the Cornea

### 3.1. Corneal-Derived EVs

Since the cornea plays a critical role in vision, it is imperative that it remains transparent and uninjured. Unfortunately, there are many environmental factors and diseases that threaten the integrity of this tissue. Studies have investigated various cellular components and processes that keep the cornea healthy. One process that has been explored is the properties and functions of EVs as mediators of cell–cell communication in the cornea. Table 1 provides a summary of all corneal diseases and their EV therapeutics discussed in this review.

EVs derived from the cornea have been shown to influence corneal wound healing. Yeung et al. found that CD81 expression was similar between corneal epithelial cells and corneal stromal cells [121]. The authors measured the size and surface potential of epithelial and stromal EVs and found no significant differences between these two groups [121]. They also showed subtle differences in the EV protein expression between epithelial and stromal cells alluded to unique phenotypes [121].

EVs secreted by the corneal epithelium increase alpha-smooth muscle actin (α-SMA) expression by keratocytes, with the epithelial basement membrane limiting their travel into the stroma [103,122,123]. An in vitro study revealed that exosomes released from cultured corneal epithelial fibroblasts and endothelial cells promote epithelial cell migration via activation of the MAPK and JAK/STAT pathways [124]. EVs derived from corneal epithelial cells have been found to contain Thrombospondin-1 (TSP-1) as well as fibronectin, which are provisional matrix proteins that influence cell contractility and migration [125]. This work suggests that EVs derived from the epithelium may be important mediators of corneal scarring and provides some evidence for a protective effect of an intact basement membrane in blunting scar formation by inhibiting EV diffusion from the epithelium following mild debridement.

It has been shown that corneal fibroblasts secrete EVs rich in MMP-2 and 14, which may be taken up by vascular endothelial cells and play a role in corneal neovascularization [126]. Parekh et al. showed that human corneal endothelial cell-derived EVs decrease the proliferation rate and wound healing response in vitro and ex vivo of endothelial cells in human, porcine, and rabbit models, as well as an increase in the cell size and apoptosis [127].

Studies have also shown that EVs derived from other origins may have beneficial effects on the cornea as well. Platelet-derived EVs (PEV) have been shown to decrease blood vessel formation in an injured cornea in an alkali-burn mouse model [128]. PEVs can also increase the wound closure rate of murine corneal endothelial cells in vitro [128]. Serum-derived EVs have been found to promote human corneal epithelial cells during an in vivo scratch assay [129]. Milk-derived EVs have been shown to increase the proliferation and migration rate of corneal epithelial cells after an alkali burn in vitro [130]. Salivary EVs have also been found to increase the proliferation and migration rate of corneal epithelial cells, both in vitro and in vivo, in a mouse model [131].

In terms of other bioactive membrane-bound particles in the cornea, extracellular mitochondria derived from injured sensory nerve axons are readily taken up by corneal epithelial cells via macropinocytosis and appear to be respiratory-competent [132]. The intercellular transport of mitochondria has been studied in different cell types in the body, including astrocytes and neurons [133], adipocytes [134], cancer, and immune cells [135] suggesting that this interplay may provide a conserved mechanism for preserving metabolic function and cell survival in times of stress [136]. Engulfed mitochondria by the corneal epithelium have been found to accelerate wound healing in a chemical burn model, providing supporting evidence that endocytosed mitochondria activate pro-reparative signaling responses [137]. Uptake of exogenous mitochondria by corneal endothelial cells in vitro has shown similar findings in a Fuch’s corneal endothelial model, with a reduction in oxidative stress and an increase in the mitochondrial membrane potential as a measure of energy production [138]. While extracellular mitochondria appear to retain respiratory function, it remains unclear if transferred mitochondria fully integrate into the host cell and remain respiratory competent in the long term.

#### EVs and Corneal Immune-Privilege Interplay

As mentioned, the cornea is an immune-privileged tissue that rapidly clears foreign bodies that enter. Resident EVs are allowed to play their role and do not trigger the cornea’s immune response. When the eye is injured, the resident immune cells are activated to remove the irritant from the eye. If treated with EVs from an external source, the cornea may work to clear the foreign EVs from the area.

Corneal grafts are an effective treatment to restore a person’s eyesight, but unfortunately, there is a high rate of graft rejection in conditions associated with inflammation or infection [139]. Rejection may be exacerbated by the introduction of EVs from an external source, which contain foreign antigens [140,141,142]. These antigens can induce inflammation and accelerate EV clearance by activating the innate and adaptive immune response [140,141,142]. This immune system activation can then lead to graft rejection in high-risk cases [140,141,142].

### 3.2. Stem Cell-Derived EV in the Corneas

EVs can be derived not only from established cell types but also from multipotent stem cells, which have the potential to serve as therapeutics for corneal injuries. Wang et al. found that induced pluripotent stem cell (iPSC)- and mesenchymal stem cell (MSC)- derived exosomes facilitate corneal wound closure after injury by increasing cell proliferation and migration. Their data suggested that iPSC-derived exosomes have a stronger therapeutic effect compared to MSC-derived exosomes [96,143]. MSCs can also be used to prolong corneal transplant graft survival in rats [143]. Shojaati et al. found that corneal stromal stem cells (CSSC)-derived EVs may play a crucial role in inhibiting corneal scarring and initiate regeneration of transparency to wounded corneal tissue [102].

MSCs derived from different sources can also provide beneficial effects to the cornea. Yu et al. introduced EVs derived from bone marrow-derived MSCs (BMSCs) to wounded epithelial cells on a cornea-on-a-chip and found that these EVs accelerated corneal epithelial wound healing [99]. Saccu et al. reported similar findings by treating wounded human corneal epithelial cells with BMSC-derived EVs and found that the cells recovered faster with modulation of angiogenesis, cell death, and inflammation in a wounded cornea [95]. BMSCs-derived exosomes can also be used as a vehicle to transport specific miRNAs to an injured cornea [143]. Adipose MSC (ASC)-derived exosomes have also been shown to have beneficial effects on rat corneal endothelial cells that underwent a freeze injury [144]. Tao et al. investigated the effects of EVs derived from human placenta-derived MSCs for alkaline injuries in a mouse model. They found that the hp-MSC EVs provided an ameliorative effect on the injured cornea by increasing anti-inflammation and proliferation, and decreasing apoptosis in the epithelial cells [101]. Hp-MSCs also provided ameliorative effects of inhibited inflammation and angiogenesis in the affected cornea [101].

### 3.3. Corneal Fibrosis

Corneal fibrosis, following injury, arises from the excessive accumulation of aberrant extracellular matrix produced by myofibroblasts [97,98]. This condition compromises the cornea’s transparency and structural integrity, significantly altering light-scattering properties and consequently reducing visual acuity [100,145].

In the cornea, communication between the corneal epithelium and stroma is critical for corneal wound repair, and EVs have been shown to influence physiological and pathological responses during this process [122]. Corneal wounds induce the release of EVs in rat epithelial debridement and rabbit keratectomy injury models [105]. While EVs released from the corneal epithelium after debridement were obstructed from entering the stroma by Bowman’s layer, a keratectomy wound, which involved the removal of Bowman’s layer and the anterior stroma, permitted the diffusion of EVs into the stromal tissue [105]. Mouse epithelial cell-derived EVs have also been reported to induce mouse corneal fibroblast proliferation and differentiation into myofibroblast [103,105]. Mesenchymal stem cells from corneal stromal stem cells-derived EVs have been reported to inhibit corneal scarring during wound healing in mice [102]. Human saliva-EVs have also been observed by our group to regulate stromal cell migration and wound healing [112]. Overall, these findings suggest that EVs may serve as a novel therapeutic target or be applied as therapeutic carriers of cargo that could prevent scar formation during the corneal wound healing process.

### 3.4. Keratoconus

Keratoconus (KC) is a corneal disease that forms the cornea into a cone shape [146,147]. It affects 1:400–1:2000 [104,147] individuals worldwide and is known to appear in puberty, extending through the third and fourth decades of life. Some common techniques to diagnose KC are by using slit lamp and tomography, but these techniques require physical manifestations to be present in order to make a diagnosis. Studies have shown molecular changes in this disease with altered levels of hormones and inflammatory factors in the blood [111,148,149,150], tears [111,148,151,152], and saliva [111,148,153] of KC patients. EVs have also been observed to have unique differences between healthy and KC samples.

Hefley et al. analyzed tear-derived EVs from healthy and KC donors. The authors found that the size and total particle counts did not differ between healthy and KC tEVs [107]. They also found that KC males showed a significant downregulation of CD63/CD9 and CD63/CD81/CD9 compared to healthy males [107]. Additional studies for KC have found that the protein cargo from corneal stromal cell-derived EVs altered levels of adhesion and migration proteins [110] as well as an overexpression of miR-184 [154]. Lozano et al. found a decreased expression of CD9 in corneal stromal cells, which suggests that KC individuals produce fewer EVs compared to healthy individuals [110]. EVs from the fibroblasts of patients with KC have been shown to contain miRNAs that have the role of silencing apoptosis, which has been found to be absent in healthy fibroblast EVs [106,155].

Escandon et al. introduced salivary EVs to human keratoconic stromal cells (HKCs) in an attempt to achieve therapeutic results in KC. They found that the salivary EVs were able to reduce the fibronectin protein expression as well as increase the thrombospondin-1 and cleaved vimentin [112]. These results support that salivary EVs could be a potential therapeutic treatment for KC.

Common treatments for this disease include collagen crosslinking, which strengthens the cornea by relinking the collagen fibrils in the cornea, and corneal transplantation (keratoplasty). One research group conducted a clinical trial to treat KC using autologous adipose tissue-derived mesenchymal stem cells (MSC(AT)). There were no post-operative complications, along with improved uncorrected and corrected distance in visual acuity at the 1- and 3-year follow-ups [36]. Considering KC as one of the leading causes of visual impairment in the cornea, more EV studies are needed in order to find potential therapeutic markers as well as to establish a biomarker for this disease.

### 3.5. Diabetes

Diabetes Mellitus (DM) is a metabolic disorder that is recognized as a major global disease, causing structural and functional ocular complications [108,156]. The prevalence of DM is increasing rapidly, contributing to a corresponding rise in corneal complications. The cornea is one of the most densely innervated and metabolically active tissues in the body. Hyperglycemia disrupts cellular and extracellular balance, leading to diabetic keratopathy. Primary complications associated with diabetic keratopathy include delayed corneal wound healing, loss of corneal sensitivity, reduced epithelial thickness, oxidative stress, and inflammation [108,109,156,157].

Roles of EVs have been identified in nearly every compartment of the eye, including the aqueous humor, vitreous humor, retina, and corneal microenvironment. EVs are essential for mediating cell–cell communication, signal transduction, and extracellular matrix remodeling in the cornea [125]. Due to its exposed location, the cornea is highly susceptible to injuries and epithelial breakdown. EVs, which are secreted by nearly all cells in the body, hold potential as therapies for treating corneal diseases owing to their key role in cell-to-cell communication [143]. Damage to epithelial cells triggers migration to heal the wound area, a process accelerated by corneal-derived exosomes and contributions from other layers [103,105,124]. Studies have shown that EVs are secreted in the cornea following injury [105,123,158]. EVs from isolated human corneal epithelial cells promote myofibroblast differentiation by corneal fibroblasts, suggesting that Bowman’s layer may provide an important barrier to pro-fibrotic EVs following wounding [103]. Supporting this idea, corneal injuries with an intact basement membrane are not generally associated with scarring compared to penetrating injuries, which occur with permeation of EVs and soluble factors into the stroma from the corneal epithelium and tear film [159].

Apart from their healing role in the cornea, EVs have been found to be significant in maintaining retinal cell function and homeostasis [160]. The role of EVs has been investigated in diabetic retinopathy (DR), which is a complication of diabetes that damages retinal blood vessels and the light-sensitive layer at the back of the eye. Studies have observed elevated levels of EVs due to inflammation, oxidative stress, and high blood glucose levels [160,161]. The potential of EVs as biomarkers has been explored in DR, where higher plasma EV numbers were observed in severe stages compared to mild cases [162]. Zhu et al. suggest that this difference in EV numbers may provide predictors for assessing the severity of DR in patients [160]. EVs also act as crucial mediators in the progression of DR, contributing to endothelial dysfunction, blood-retinal barrier breakdown, and neovascularization. In addition to this, EVs carry miRNAs, proteins, and nucleic acids that trigger pro-inflammatory and pro-angiogenic cascades, contributing to retinal damage [163]. Additionally, EVs have been investigated for their role in retinal pigment epithelium (RPE), where they induce an immunoregulatory CD14++CD16++ phenotype that inhibits T-cell proliferation without affecting cell survival [163].

Several studies have explored the therapeutic benefits of exogenous EVs as therapeutics in pre-clinical models of diabetes. Hefley et al. investigated EVs derived from healthy and diabetic corneal stromal constructs, revealing larger sizes and higher total particle counts in EVs derived from healthy constructs compared to diabetic ones, suggesting that diabetes may be associated with differential EV-mediated signaling in the corneal stroma [114]. Therapeutic applications of EVs derived from mesenchymal stem cells from adipose tissue have been demonstrated, showing corneal nerve regeneration and sensation recovery in streptozotocin (STZ)-induced diabetic keratopathy (DK) mice, correlating with activation of pro-reparative responses [116]. EVs have also been found to significantly promote corneal epithelial wound healing through activation of Nerve Growth Factor (NGF)/Tropomyosin receptor kinase A (TrkA) pathway, which is an essential pathway involved in the maintenance of corneal sensory nerves that penetrate the epithelium and stroma [116]. Studies involving EVs derived from healthy corneal epithelial cells, MSCs, or limbal stem cells show potential in reversing diabetic corneal damage through restoration of normal epithelial wound healing, reduction of oxidative stress and inflammation, and delivery of beneficial miRNAs to corneal cells. This area can be further explored to better understand the role of EVs in corneal diabetes, along with other corneal diseases.

### 3.6. Herpes Simplex Virus

The Herpes Simplex Virus (HSV) is a common virus that can be transmitted through bodily fluids and can cause inflammation of the cornea and loss of corneal innervation. HSV type 1 (HSV-1) can lead to corneal infection, which can cause the infected individuals to have Herpes Simplex Keratitis (HSK), which is the leading factor for infectious blindness [118]. HSV-2 can cause lesions on the genital area. HSK can travel to the trigeminal ganglion after the first exposure and remain latent. Once activated, HSK can affect the epithelium, stroma, and endothelium layer of the cornea, in which recurring infections can lead to corneal thinning, neovascularization, and scarring of the corneal epithelium [118]. This disease can also affect other areas of the eye, such as the iris, lens, vitreous humor, and retina, which leads to iridocyclitis, anterior uveitis, vitritis, cataracts, and retinal detachment if not caught in time.

Slit lamp evaluations are the first step in diagnosing HSK. If the slit lamp evaluation is inconclusive, the clinician may take a sample and perform PCR for a confirmation test. Current treatments for HSK include topical antibacterials and antivirals.

Little is known about EVs in the study of HSK. Ma et al. identified 339 metabolites from tear EVs collected from patients with HSK and found altered levels of amino acid and energy metabolism, providing insight into the effects of HSV on metabolic activity in host cells [118].

Even though HSV is one of the more prevalent sexually transmitted diseases, the spread of HSK is not entirely known. A recent study suggests that tear exosomes could be the latent site of the HSV-1 virus in recurring HSK, which could lead to transmission of this virus. This study confirmed that HSV-1 genes can be transferred intercellularly by the exosomal pathway, highlighting EVs as both biomarkers and potential mediators of infection [120].

### 3.7. Dry Eye Disease

The cornea is protected from environmental factors, such as dirt and dust, by the tear film, which also keeps the cornea moist. The tear film is comprised of three layers: the lipid layer, aqueous layer, and finally the mucin layer. In a healthy individual, this film is equally spread across the anterior of the eye and is replaced quickly after each blink. Individuals with dry eye have a reduction in this tear film, which will lead the affected individual to experience itching, glare, eye irritation, blurry vision, and discomfort. There are two different types of dry eye. The first type is evaporative dry eye (EDE), in which the mucosal layer is diminished in the tear film, leading to the evaporation of the aqueous layer. The second type of dry eye is aqueous tear deficiency (ATD), in which not enough of the aqueous layer is produced.

Studies are hoping to determine whether EVs can be used as a diagnostic tool to identify dry eye and to decrease the number of tests needed to diagnose an individual with dry eye. Studies evaluating small RNA cargo in EVs have revealed notable differences in microRNAs in dry eye disease. Cross et al. analyzed RNA transcripts from dry eye EVs and found 26,000+ different transcripts [48]. Of these transcripts, 6% showed significantly altered levels, such as sodium channel modifier 1 (SCNM1) and microRNA-130b, between healthy individuals and individuals with dry eye [48]. Pucker et al. collected tear samples from healthy and dry eye individuals and found an upregulation of inflammatory markers, including miR-127-5p, miR-1273h-3p, miR-1288-5p, miR-139-3p, miR-1910-5p, miR-203b-5p, miR-22-5p, miR-4632-3p, and miR-130b-5p, which is what Cross et al. also observed [113].

The current treatment for dry eye is artificial tears, which only provides a temporary solution. Current research is being conducted in search of a better therapeutic option for this disease. Yu et al. used a dry-eye mouse model, which induced corneal damage, to test the therapeutic effects of EVs derived from human adipose tissue stem cells (hADSC-EVs). They found that eye drops containing hADSC-EVs effectively alleviate ocular surface damage by decreasing the NLRP3 inflammatory response in dry eye [115]. Yi et al. investigated EVs derived from human amniotic epithelial cells (hAEC-EVs) and observed decreased inflammation in the ocular surface in an in vivo mouse model [117]. Ocular surface epithelial cells treated with hAEC-EVs had reduced inflammatory cytokines, as well as reduced corneal epithelial cell migration and proliferation [117]. Wang et al. found that when a mouse model that has dry eye is treated with EVs derived from human umbilical cord-derived MSCs (huMSC-EVs), the IRAK/TAB2/NF-κB pathway is targeted, reducing inflammation and restoring the corneal surface [119].

### 3.8. Bioengineering Applications

One of the challenges that limits the therapeutic application of EVs introduced to the cornea has the potential to be rapidly cleared due to the immune-privileged properties of the cornea, such as tear turnover, blinking, nasolacrimal drainage, and limited epithelial permeability [164,165]. Hydrogels, with their features including biocompatibility, high water content, and adjustable mechanical properties, help sustain the release of corneal epithelial cell-derived EVs [166,167,168]. Tang et al. have also implemented iPSC-MSC-derived EVs into hydrogels and have observed a similar sustained EV release in a damaged cornea [169]. The iPSC-MSC-derived EVs have been observed to reduce scar formation in vivo by decreasing the mRNA expression that codes for collagen type 1 alpha 1, collagen type V alpha 1, and collagen type V alpha 2, which are highly expressed collagens in the cornea [169]. Additionally, studies on biopolymer-based hydrogels loaded with MSC-derived exosomes demonstrate increased corneal adhesion and sustained bioavailability in vivo, further supporting the application of a hydrogel delivery platform for improving EVs residence time in corneal applications [170,171]. In corneal epithelial injury models, Sun et al. showed that exosome-loaded DEGMA-modified hyaluronic acid hydrogel stayed on the corneal surface much longer than free exosomes and enhanced prolonged local exposure of miRNA cargo on the ocular surface, which enhanced epithelial regeneration and reduced inflammation [172].

### 3.9. Challenges in Clinical Translation

As we have presented in this review, there are many sources and methods used to introduce EVs to the cornea. There is currently no standardization of EV research in place, which may lead to varying results between labs using the same techniques; therefore, there is a need for standardization practices in this field. MISEV is attempting to provide standardization techniques to scientists in the field to ensure uniformity in EV research. They also describe terminology to spread awareness that there are not only three types of EVs, but numerous types of vesicles and particles present in samples.

Another challenge that needs to be overcome in moving forward with using EVs as therapeutics is the manufacturing processes, as well as the stability of these vesicles. EVs are produced from cells, so there needs to be quality control to ensure they are following the MISEV guidelines. EVs are also produced from cells, which can be a challenge in itself since cells are a precious resource. EVs also have to be stored at a certain temperature to maintain stability, and freeze-thaw cycles may impact their characteristics and effectiveness. The EVs would also have to contain self-antigens to avoid detection by the cornea’s immune system.

## 4. Conclusions

EVs could be used as a powerful tool to treat corneal diseases since it has been suggested that they have a beneficial impact on the wound-healing cascade. Since EVs are derived from cells and can be found in biological fluids, they may provide valuable insight into the health of the cornea by utilizing them as biomarkers for various corneal diseases. EVs can also serve as a natural drug delivery system, which can bypass the body’s immune system.

There are numerous challenges that must be overcome for EVs to be safe to use as therapeutics. Some of these challenges include EV heterogeneity, the immune-privileged properties of the cornea, delivery of EVs, and mass-production methods, to name a few. Further research on EVs is warranted to determine their use as biomarkers, as well as therapeutic options for the corneal diseases described in this review.

## Figures and Tables

**Table 1:** Summary of corneal EV therapeutics.

Disease	EV Source	Findings	Reference
Corneal wound healing and fibrosis	iPSCs and MSCs	accelerated corneal epithelial wound healing	[95,96]
	Bone marrow-derived MSCs	accelerated corneal epithelial wound healing	[95,97,98,99]
	placenta-derived MSCs	beneficial effect on corneal epithelial wound healing	[100,101]
	MSCs from corneal stromal stem cells	Reduced murine corneal scarring	[101,102]
	Human corneal epithelial cell	Drives the differentiation of primary human corneal fibroblasts into myofibroblast	[103]
	Mouse Corneal epithelial cell	mediate differentiation of mouse corneal keratocytes into myofibroblast	[104,105]
Keratoconus (KC)	Tear from healthy and KC donors	KC males showed a significant downregulation of CD63/CD9 and CD63/CD81/CD9 compared to healthy males	[106,107]
	Healthy and KC primary human cornea stroma cells	Alteration in KC primary human cornea stroma cells-derived exosomes.	[106,108,109,110]
	Saliva	reduce the fibronectin protein expression as well as increase the thrombospondin-1 and cleaved vimentin in the KC primary human stroma fibroblast.	[111,112]
Diabetic keratopathy	co-culture construct of primary human corneal fibroblasts and immortalized human corneal epithelial cells	Healthy constructs produced more and larger EVs compared to diabetic constructs.	[113,114]
	Mouse adipose-derived MSCs	Facilitate diabetic corneal epithelial wound healing through NGF/TrkA pathway activation.	[115,116]
Herpes Simplex Keratitis (HSK)	Tear from healthy and HSK donors	Altered levels of amino acid and energy metabolism in EVs from HSK donors.	[117,118]
	Tear from healthy and HSK donors	Tear exosomes may be the latent sites of HSV-1 in recurrent HSK and might be involved in the spread of HSV-1.	[119,120]
Dry Eyes Diseases (DED)	Tears from a group with a tear film break-up time (TBUT) of 2 s and 10 s	A total of 26,639 different RNA transcripts were identified, with 6% showed significantly altered levels between groups with TBUT 2 s and the group with TBUT 10 s.	[48]
	Tears from healthy and DED donors.	Tear-derived EVs contain miRNAs that may be associated with DED inflammatory pathways	[113]
	Adipose tissue stem cells (hADSC)	Topical hADSC-EVs protected the ocular surface by suppressing NLRP3 inflammasome activation in DED mice.	[115]
	Human amniotic epithelial cells	Increased corneal epithelial cell proliferation and migration, and reduced inflammatory cytokines. Improved tear production and reduced ocular surface inflammation in DED mice.	[117]
	Human umbilical cord-derived MSC	Increased tear volume and reduced inflammation by suppressing the IRAK1/TAB2/NF-κB pathway in DED mice.	[119]

## Data Availability

No new data were created or analyzed in this study.

## Abbreviations

The following abbreviations are used in this manuscript:

EV	Extracellular Vesicles
ECM	Extracellular Matrix
NCAM	Neural Cell Adhesion Molecules
NTA	Nanoparticle Tracking Analyzer
PCR	Polymerase Chain Reaction
α-SMA	Alpha Smooth Muscle Actin
PEV	Platelet-derived EVs
iPSCs	Induced Pluripotent Stem Cells
MSCs	Mesenchymal Stem Cells
CSSCs	Corneal Stromal Stem Cells
BMSCs	Bone Marrow-derived MSCs
ASCs	Adipose MSCs
KC	Keratoconus
HKC	Keratoconic Stromal Cells
MSC(AT)	Autologous Adipose Tissue-derived MSCs
DM	Diabetes Mellitus
DR	Diabetic Retinopathy
RPE	Retinal Pigment Epithelium
STZ	Streptozotocin
DK	Diabetic Keratopathy
NGF	Nerve Growth Factor
TrkA	Tropomyosin Receptor Kinase A
HSV	Herpes Simplex Virus
HSV-1	Herpes Simplex Virus Type 1
EDE	Evaporative Dry Eye
ATD	Aqueous Tear Deficiency
SCNM1	Sodium Channel Modifier 1
hADSC-EVs	Human Adipose Tissue Stem-Cells derived EVs
hAEC-EVs	Human Amniotic Epithelial Cells-derived EVs
huMSC-EVs	Human Umbilical Cord-derived MSCs-derived EVs
